# Salt-inducible kinase 1 is a key gene in suppressing EVD68-induced asthma by modulating antiviral immunity

**DOI:** 10.1016/j.gendis.2025.101845

**Published:** 2025-09-10

**Authors:** Juntong Liu, Yue Wang, Lingyun Zou, Xinyue Han, Mingqi Lv, Xichuan Deng, Jingjing Liao, Guangchao Zang, Lei Xu, Tianle Gu, Nan Lu, Guangyuan Zhang

**Affiliations:** a Pathogen Biology and Immunology Laboratory, Lab Teaching & Management Center, School of Basic Medical Sciences, Chongqing Medical University, Chongqing 400016, China; b Department of Clinical Data Research, Chongqing Emergency Medical Center, Chongqing University Central Hospital, Chongqing University, Chongqing 400014, China

**Keywords:** Antiviral immunity, Asthma, EV-D68, SIK1, Virus

## Abstract

Asthma is a complex inflammatory disease of the airways, affecting over 300 million individuals globally. Infection with enterovirus D68 (EV-D68) has been identified as a risk factor for asthma. However, the biological mechanisms of EV-D68-related asthma remain unclear. In this study, using machine learning techniques, we identified salt-inducible kinase 1 (SIK1), which plays a crucial role in associating with the asthma phenotype and EV-D68 infection. Concretely, a negative correlation between SIK1 expression and asthma risk has been revealed through Mendelian randomization. Immune infiltration analyses showed that SIK1 was negatively correlated with mast cell activity and positively correlated with T cell responses. Using weighted gene co-expression network analysis, we demonstrated SIK1's role in antiviral immune responses in asthma. Further *in vitro* and *in vivo* experiments confirmed that SIK1 was up-regulated in virus infection, and it exerted antiviral effects in various viral infections. Finally, in the asthma exacerbation model of HDM combined with EV-D68 infection, SIK1 activation effectively mitigated EV-D68-induced asthma exacerbation in mice. Taken together, our findings suggest that SIK1 serves as a protective factor in EV-D68-induced asthma by modulating antiviral immune responses, which provide new insights into potential treatments for EV-D68-induced asthma attacks.

## Introduction

Recent global outbreaks of respiratory viral infections have highlighted the critical public health concern of the relationship between viruses and chronic respiratory diseases.^1^ Asthma, a prevalent and complex inflammatory airway disease, affects over 300 million people globally.^2^ The immunology of asthma is characterized by airway inflammation and remodeling, driven by the persistent accumulation and interaction of inflammatory cells and mediators.^3^ Research suggests that respiratory viral infections, such as respiratory syncytial virus, rhinovirus, and human parainfluenza virus type 3, are important triggers of asthma exacerbations.^4^^,^^5^

EV-D68, which belongs to group D of the *Enterovirus genus*,^6^ has been circulating and endemic in several locations worldwide since 2014.^7^ Unlike other enteroviruses, EV-D68 primarily causes respiratory disease and can lead to symptoms such as respiratory distress and severe bronchospasm requiring intensive care.^8^ A robust correlation between the prevalence of EV-D68 and the occurrence of asthma has been demonstrated by epidemiological studies. During the 2014 EV-D68 outbreak in the United States, 52% of patients (304) had a history of asthma or reactive airway disease, with 66% requiring intensive care.^9^ A 2015 study in Japan linked a surge in childhood asthma hospitalizations to EV-D68 prevalence.^10^ Similarly, in 2018, 53% of 277 EV-D68 patients admitted to Nationwide Children's Hospital in Ohio had a history of asthma.^11^ Additionally, molecular epidemiological and clinical control studies have demonstrated that children infected with EV-D68 are more prone to having a history of asthma and developing acute respiratory illness, particularly persistent wheezing, in comparison to children with other rhinovirus infections.^12^

Prior research has identified substantial contributions of host genes to the development of asthma induced by viral infections. For instance, cullin 5 (CUL5) has been demonstrated to contribute to the development of asthma by suppressing antiviral immunity and type I interferon production, while simultaneously promoting neutrophil accumulation.^13^ Conversely, gasdermin B (GSDMB) has been shown to enhance interferon-stimulated gene (ISG) expression and airway inflammation during respiratory syncytial virus infection, thereby increasing the risk of developing asthma in individuals who carry the risk allele.^14^

Nevertheless, despite the evident epidemiological evidence indicating that the EV-D68 virus is a trigger and exacerbator of asthma, the precise molecular mechanisms and the role of host genes in this process remain unreported. Consequently, there remains no means of addressing asthma caused by EV-D68 infection, although novel biotherapies that target host genes rather than the virus itself present novel avenues for intervention and personalized treatment of severe asthma.^3^

This study aims to integrate bioinformatics and experimental research methods to explore host genes that play pivotal roles in EV-D68 infection and asthma. Through machine learning, Mendelian randomization (MR), immune infiltration, weighted gene co-expression network analysis, and validation of *in vivo* and *in vitro* experiments, we show that salt-inducible kinase 1 (SIK1) plays a protective role in EV-D68-induced asthma exacerbation by modulating the antiviral immune response. Our findings propose an important function for SIK1 in virus-induced asthma exacerbations, indicating SIK1 as a target for asthma exacerbation treatment.

## Materials and methods

### Data sources

The datasets employed in this study were procured from the Gene Expression Omnibus database and encompass expression profiling data pertaining to EV-D68 infection, asthma, and other viral infections. Specifically, the GSE184488 dataset comprises RNA-sequencing data from EV-D68-infected respiratory tract organoids and control samples. The GSE143303 dataset comprises microarray expression profiling data from bronchial biopsy samples of 47 asthma patients and 13 healthy subjects, using the GPL10558 platform. The GSE152004 dataset comprises RNA-sequencing data from nasal epithelial rinse samples of 441 patients with asthma and 254 healthy subjects. The GSE65204 dataset contains microarray data (GPL14550) from nasal epithelial rinse samples of 36 asthma patients and 33 healthy subjects. The GSE157103 dataset comprises whole blood RNA-sequencing data from 100 patients diagnosed with COVID-19 and 26 healthy subjects. The final dataset, GSE243200, comprises RNA-sequencing data from seven-day mouse thymus samples from five sets of coxsackievirus A6 (CA6) infection and five sets of control samples.

### Differential expression gene screening

The GSE143303 dataset was subjected to differential expression analysis using the “limma” package in the R environment. Because this dataset was derived from clinical samples, the threshold for identifying differentially expressed genes (DEGs) was set at a *p*-value of less than 0.05 and |log_2_fold-change| ≥ 0.2. Additionally, the GSE184488 dataset underwent differential expression analysis using the DESeq2 package, with the threshold for identifying DEGs set at a *p*-value of less than 0.05 and |log_2_fold-change| ≥ 1.

### Functional enrichment analysis

Gene Ontology (GO) and Kyoto Encyclopedia of Genes and Genomes (KEGG) enrichment analyses were conducted using the cluster Profiler package to elucidate the functions and pathways associated with DEGs in the EVD-68 infection and asthma datasets. The GO terms identified from the GO enrichment analysis facilitated comprehension of the regulatory relationships between genes within cellular components (CC), molecular functions (MF), and biological processes (BP). The KEGG enrichment analysis revealed multiple disease pathways involving these genes and provided annotations of their functions. Gene Set Enrichment Analysis (GSEA) was performed on gene expression data using the cluster Profiler package in R, with the significance of gene sets across different phenotypes assessed through enrichment scores and adjusted *p*-values.

### Signature gene identification

A LASSO regression model was constructed using the tenfold cross-validation method (glmnet package) to test and eliminate redundant genes. Concurrently, the Support Vector Machine-Recursive Feature Elimination (SVM-RFE) method was employed to assess the potency of the genes and select the optimal subset. The intersection of the gene subsets obtained from the LASSO regression and SVM-RFE methods was identified using Venn diagrams. Subsequently, the overlapping genes were ranked in order of importance using the Random Forest and XGBOOST algorithms.

### Immune infiltration analysis

An immune infiltration analysis was conducted using the CIBERSORT algorithm to determine the proportions of 22 infiltrating lymphocyte subpopulations in both asthma and healthy samples. Pearson correlation coefficients were subsequently applied to evaluate the correlations between each type of immune cell and between immune cells and labeled genes.

### Two-sample MR analysis of signature gene expression and asthma phenotypes

An MR approach was employed to assess the causal relationship between tagged gene expression and asthma risk using the R package Two Sample MR. The eQTL data pertaining to SIK1 expression were initially extracted from the Integrative Epidemiology Unit (IEU) database (SIK1 ID: eqtl-a-ENSG00000142178). The instrumental variables were screened from the eQTLs according to the following criteria: a significance threshold of *p*1 = 5e-05, a linkage disequilibrium parameter of r2 = 0.3, and a gene window size of 100,000 bp. These rigorous criteria guaranteed the statistical reliability of the chosen instrumental variables while reducing the likelihood of false positives due to linkage disequilibrium. Subsequently, the instrumental variables were subjected to two-sample MR analysis using data from a genome-wide association study of asthma (ebi-a-GCST90018795). Sensitivity analyses were conducted to verify the genetic heterogeneity and stability of the MR hypothesis.

### Weighted gene co-expression network analysis

To investigate the biological mechanisms of SIK1 in respiratory tissues, weighted gene co-expression network analysis (WGCNA) was conducted on the variance-stabilizing transformation (VST) matrix of 17,473 expressed genes in the GSE152004 dataset. In the analysis parameters, the soft threshold β was set to 13, the min Cluster Size was set to 20, the deep Split was set to 1, and the pam Stage was set to TRUE. A total of 36 co-expression networks were identified and characterized. Subsequently, GO and KEGG enrichment analyses were performed to identify the biological processes and signaling pathways associated with these networks.

### Cells, viruses, siRNAs, and plasmids

Human rhabdomyosarcoma (RD), HeLa, and A549 cell lines were kept in our laboratory and cultured in Dulbecco's Modified Eagle Medium (DMEM, GIBCO) supplemented with 10% fetal bovine serum (BI), 1% penicillin/streptomycin (HyClone, USA) at 37 °C in a humidity incubator with 5% CO_2_.

The EV-D68 (ATCC VR-1826), EV-A71, HSV-1, and VSV-GFP were kept in our laboratory. The viruses EV-D68 and EV-A71 were propagated in RD cells, and the viruses HSV-1 and VSV-GFP were propagated in Vero cells, all through inoculation at a multiplicity of infection of 1.

Small interfering RNAs (siRNAs) specific for SIK1 (siSIK1) were synthesized by Sangon Biotech (Shanghai, China), and sequences are presented in Table S1. The plasmids pCDH-CMV-GFP-SIK1-Flag were purchased from Wuhan Biorun Ltd. (Wuhan, China).

### Virus infection and titer assays

Cells were seeded into 6-well plates. Upon reaching a cell density of 70%–80%, the cells were washed 3 times and incubated with the respective virus liquid. 2 h later, viruses were removed and the medium was replaced with DMEM containing 2% fetal bovine serum. Cell samples were collected at relevant time points depending on the situation. The viral titers of EV-D68, EV-A71, and human herpes simplex virus type 1 (HSV-1) were determined using 50% tissue culture infectious dose (TCID50) assays, applying the Reed–Muench formula. For the vesicular stomatitis virus-GFP (VSV-GFP), the titer was determined using a viral plaque assay. Concretely, A549 cells were seeded in 24-well plates and cultured to 80% confluence, followed by infection with a 1:10^5^ to 1:10^7^ dilution of the VSV-GFP virus. After 3 h of infection, the medium was discarded and replaced with medium containing 1.5% methylcellulose. After 48 h, the cell layers were fixed with paraformaldehyde and stained with crystal violet, and the viral titer was calculated by counting the number of plaques.

### Quantitative reverse transcription PCR

Total RNA from cells or mouse lung tissues was extracted using TRIzol reagent (LEAGENE, China). The Prime Script™ RT kit with gDNA eraser (TaKaRa, Japan) was used for reverse transcription of total RNA; reactions were initiated with 1000 ng of RNA for each sample. Relative gene expression level was determined using BioRun ChemoHS qPCR Mix (Biorun, China). Gene expression changes were calculated by the 2^−ΔΔCT^ method. Glyceraldehyde-3-phosphate dehydrogenase (GAPDH) was applied as the internal reference to normalize the target gene expression. All the primers used were synthesized in Sangon Biotech Ltd., Shanghai, China, and the sequences are listed in Table S1.

### Western blotting assay

Mouse pup tissues or cells were lysed using TNE buffer (50 mM Tris-Cl [pH 7.4], 150 mM NaCl, 2 mM EDTA [pH 8.0], 0.1% 2-mercapto-ethanol, and protease inhibitor cocktail). Protein concentration in the lysate was determined using a BCA Protein Assay Kit (Beyotime, Shanghai, China). The protein samples were separated on a 10% sodium dodecyl sulfate-polyacrylamide gel electrophoresis (SDS-PAGE) and then transferred to a PVDF membrane for western blotting detection. The membrane was blocked for 30 min with 5% skim milk in phosphate-buffered saline containing Tween 20 (1/1000) at room temperature. After that, the PVDF membrane was incubated with the primary antibody for 1.5 h with primary antibodies against EV-D68 VP1 (Sabin polio virus protein 1; GeneTex, Shanghai, China), EV-A71 VP1 (GeneTex, Shanghai, China), SIK1 (Proteintech Group, Wuhan, China), and β-actin (Proteintech Group, Wuhan, China), followed by the secondary antibody for 45 min with horseradish peroxidase-conjugated goat anti-mouse IgG (Sangon, China) or horseradish peroxidase-conjugated goat anti-rabbit IgG (Sangon, China). The protein bands were visualized using an ECL chemiluminescence kit (Beyotime, Shanghai, China).

### EV-D68 infection and treatment

Eight-to-ten-week-old type I interferon receptor-deficient C57 mice (*Ifnar*^*−/−*^) (Chongqing Medical University) were used to establish the animal model of EV-D68 infection. All the animal experiments were conducted according to the protocol approved by the Ethics Committee of Chongqing Medical University (approval number: IACUC-CQMU-2024-0669). All mice were randomly allocated to the mock and EV-D68-infected group (*n* = 3 for each group). EV-D68 (1 × 10^6^ PFU/kg in 20 μL DMEM) or mock (DMEM) was inoculated through the intranasal route after anesthesia.

### Hematoxylin-eosin staining

The mouse lung tissues were obtained for staining. After fixing in 4% paraformaldehyde for 3 days, the tissue was dehydrated and then embedded in paraffin. After the embedded tissue was sectioned into 4 μm sections with a micro-chipper and tiled on adhesive glass slides, the slices were incubated at 50 °C overnight. The tissues were stained using hematoxylin and eosin for analysis.

### EV-D68-induced asthma exacerbation model

C57BL/6 mice (6–8 weeks old, Chongqing Medical University) were randomly divided into three experimental groups that received house-dust-mite (HDM) (Greer labs, USA) sensitization alone, HDM followed by EV-D68 challenge, or HDM plus EV-D68 challenge combined with metformin (MCE, USA) treatment. On day 0, each mouse was intranasal sensitized with 250 μg/kg HDM extract dissolved in 20 μL phosphate-buffered saline (10 μL per nostril). To establish allergic airway inflammation, the same HDM dose was administered once daily on days 7–11. Viral exacerbation was induced on days 12–13 by intranasal instillation of EV-D68 (1 × 10^6^ PFU/kg in 20 μL DMEM); animals assigned to the treatment cohort received metformin intraperitoneally at 250 mg kg^−1^ (MCE, USA) once daily from day 12 through day 14. On day 15, mice were anaesthetized and tracheostomized, and broncho-alveolar lavage fluid (BALF) together with lung tissue was harvested for subsequent analyses.

### Assessment of airway hyper-responsiveness

Airway hyper-responsiveness was assessed immediately before tissue collection. Mice were intraperitoneally anaesthetized with chloral hydrate (Sigma, USA), intubated, and connected to a BL-420N biological signal-acquisition system (Taimeng, Chengdu, China). Respiratory flow parameters were recorded while animals were exposed to escalating concentrations of aerosolized acetyl-β-methylcholine chloride (Sigma, USA). Penh values were used as an index of airway hyper-responsiveness.

### Differential cell counting in BALF

Lungs were lavaged three times with sterile phosphate-buffered saline. The recovered fluid was centrifuged, and total leukocytes were enumerated in a hemocytometer. Cytospin preparations were stained with Diff-Quik (Wright–Giemsa), and at least 300 cells per slide were classified as neutrophils, eosinophils, or macrophages.

### Statistical analysis

The statistical analyses were conducted using the R (v4.2.0) language and RStudio IDE (V2022.02.2–485). All data were represented as mean ± standard deviation from at least three independent experiments. Statistical comparisons were made by unpaired Student's *t*-test (for two-group comparisons) or one-way analysis of variance (for multiple group comparisons). By default, *p* values < 0.05 were deemed to be statistically significant.

## Results

### Identification and functional annotation of co-differentially expressed genes between EV-D68 infection and asthma

**Figure 1:**
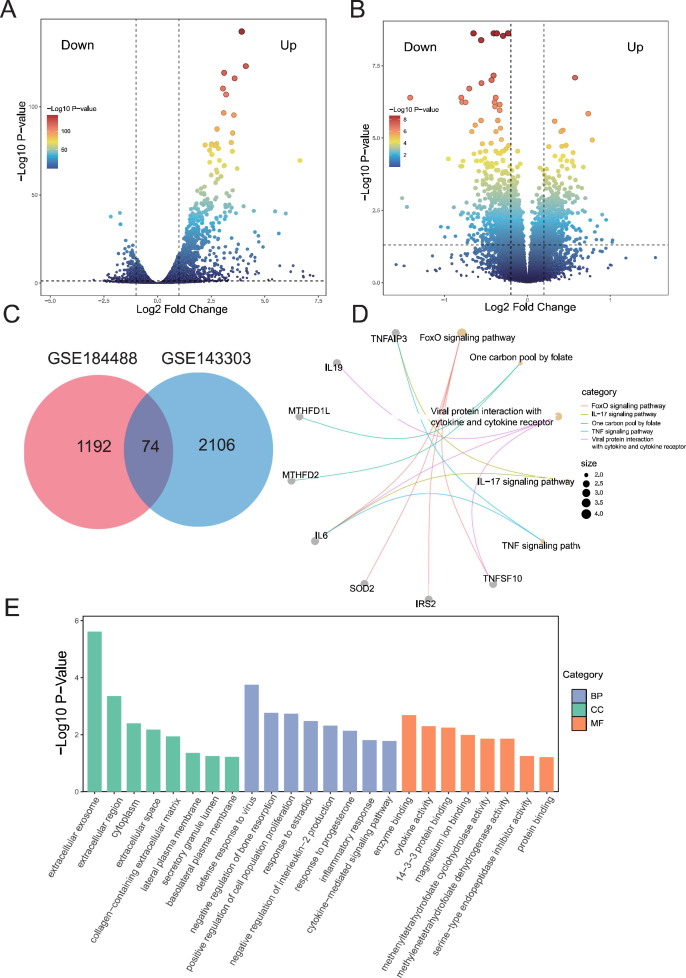
The common differentially expressed genes between EV-D68 infection and asthma. **(A, B)** Volcano plot of differentially expressed genes (DEGs) in the EV-D68-related GSE184488 dataset and asthma-related GSE143303 dataset. **(C)** Common DEGs in the GSE184488 dataset and the GSE143303 dataset were represented by Venn diagrams. **(D)** KEGG enrichment analysis of the 74 common DEGs identified in EVD68 infection and asthma. **(E)** GO enrichment analysis of the 74 common DEGs identified in EVD68 infection and asthma. BP, biological process; CC, cellular component; MF, molecular function.

A total of 1266 DEGs associated with EV-D68 infection were identified from the GSE184488 dataset (Fig. 1A), while 2180 asthma-associated DEGs were identified from the GSE143303 dataset (Fig. 1B). A total of 74 coDEGs were shared between the two groups (Fig. 1C), suggesting that they may play a potential role in the development of asthma caused by EV-D68 infection.

The KEGG pathway enrichment analysis of the 74 coDEGs revealed that they were primarily involved in the forkhead box-O (FOXO) signaling pathway, the one carbon pool by folate, the viral protein interaction with cytokine and cytokine receptor, the interleukin 17A (IL-17A) signaling pathway, and the tumor necrosis factor (TNF) signaling pathway (Fig. 1D). In the biological process enrichment analysis, the coDEGs were primarily involved in inflammatory response, cellular response to lipopolysaccharide, positive regulation of smooth muscle cell proliferation, and other processes. In the cellular component enrichment analysis, the majority of the coDEGs were situated within the extracellular exosome, the extracellular region, and other cellular components. In the molecular function enrichment analysis, the coDEGs were predominantly linked to enzyme binding, cytokine activity, and other functions (Fig. 1E).

Furthermore, GSEA KEGG pathway analysis of the datasets GSE184488 and GSE143303 demonstrated that the IL-17A pathway exhibited a notable elevation in respiratory tissues in both EV-D68 infection and asthma conditions (supplementary data; Fig. S1A, S1B). This finding suggests that the IL-17A pathway may act as a conduit between EV-D68 infection and the asthma phenotype.

### SIK1 is a signature gene for the asthma phenotype

**Figure 2:**
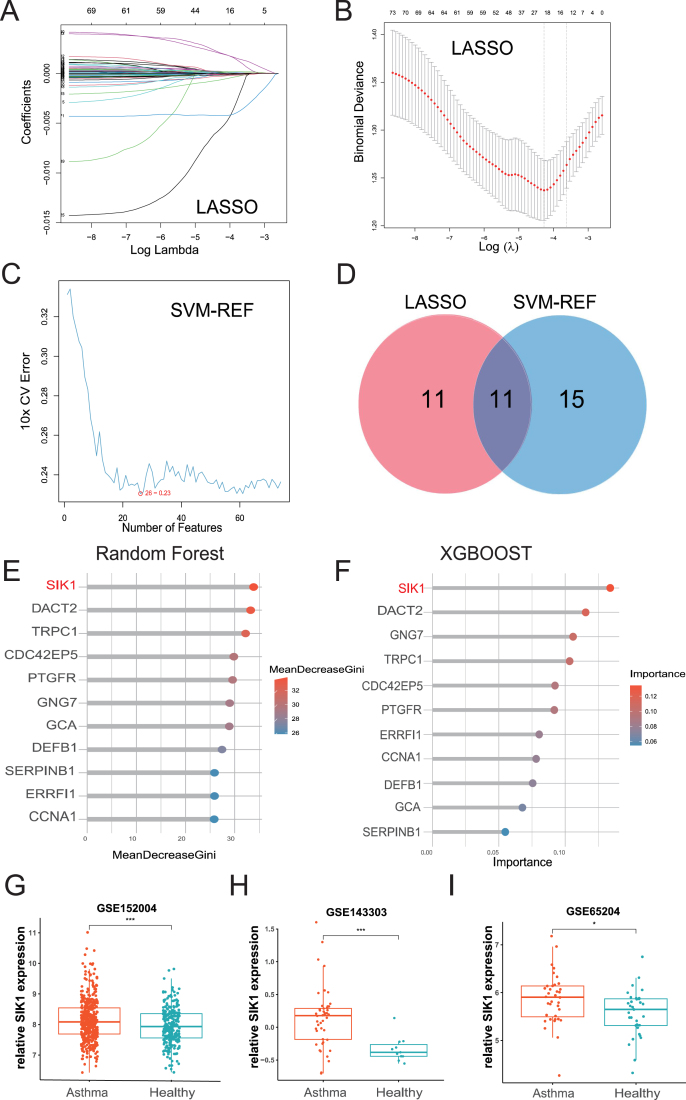
Identification of SIK1 as a signature gene in asthma by machine learning. **(A, B)** LASSO regression identified 22 key genes associated with asthma. **(C)** The SVM-RFE algorithm identified a total of 26 key genes associated with asthma. **(D)** The intersection of LASSO and SVM-RFE methods revealed a set of 11 key genes associated with asthma. **(E, F)** Using Random Forest and XGBoost to rank the importance of 11 key genes. **(G**–**I)** The expression level of SIK1 in the GSE152004, GSE143303, and GSE65204 datasets of asthma. LASSO, least absolute shrinkage and selection operator; SVM-RFE, support vector machine-recursive feature elimination; XGBoost, extreme gradient boosting.

Whole transcriptome sequencing data from 695 healthy and asthmatic children with nasal airway brushing (GSE152004) were analyzed to validate the ability of the 74 coDEGs to discriminate between diseased and healthy samples using various machine learning methods. A subset of 22 genes demonstrated the best performance in the cross-validation test of the LASSO regression model (Fig. 2A and B). In the SVM-RFE classification model, a subset of 26 genes exhibited the smallest root mean square error (RMSE) (Fig. 2C). The gene subsets identified by these algorithms were then intersected, resulting in the identification of 11 genes as potential markers: CCNA1, CDC42EP5, DACT2, DEFB1, ERRFI1, GCA, GNG7, PTGFR, SERPINB1, SIK1, and TRPC1 (Fig. 2D). Subsequently, these genes were ranked according to their importance using the Random Forest and XGBOOST algorithms. SIK1 was ranked first in both ranking groups (Fig. 2E and F).

SIK1, a serine/threonine kinase, has rarely been reported in the context of asthma. Therefore, we further validated the expression of SIK1 in multiple asthma patient cohorts (GSE143303, GSE152004, and GSE65204). The results showed that SIK1 was significantly up-regulated in both respiratory tract biopsy samples and nasal brush samples (Fig. 2G–I), indicating the credibility of SIK1 as a marker gene for the asthma phenotype.

### MR analysis reveals a negative association between SIK1 and asthma risk

**Figure 3:**
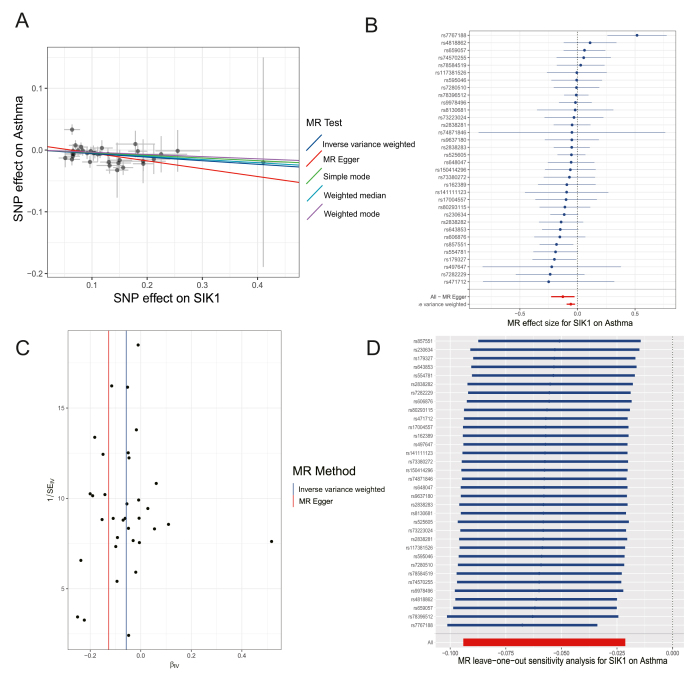
SIK1 is negatively associated with asthma risk. **(A)** The scatter plot depicts the causal impact of SIK1 on the susceptibility to asthma. **(B)** Funnel plots were utilized to assess the overall heterogeneity of MR estimates for the influence of SIK1 on asthma. **(C)** The forest plot shows the causal effect of individual single-nucleotide polymorphisms on the risk of asthma. **(D)** Leave-one-out plot was employed to visualize the causal effect of SIK1 on asthma risk when excluding one single-nucleotide polymorphism at a time.

To gain further insight into the potential relationship between SIK1 and asthma, we conducted a two-sample MR analysis. A comprehensive evaluation of all included single-nucleotide polymorphisms (SNPs) was performed to ensure the reliability of the analysis and the robustness of the results. Further details on the relevant SNP parameters can be found in Table S2. The inverse-variance weighted (IVW) analyses yielded a significant negative correlation between SIK1 and the risk of asthma, with an odds ratio of 0.943 (*p* = 0.0018) (Fig. 3A and B). The funnel plot demonstrated a roughly symmetrical distribution of causal effects (Fig. 3C). Each SNP was excluded individually, and a systematic MR analysis was re-performed on the remaining SNPs, with the results remaining consistent (Fig. 3D). All instrumental variables suggested a significant causal link between SIK1 and asthma, thereby eliminating the possibility of SNP interference and confirming the high robustness of the initial MR analysis.

### Immune infiltration analysis reveals a close association between SIK1 and asthma-linked immune cells

**Figure 4:**
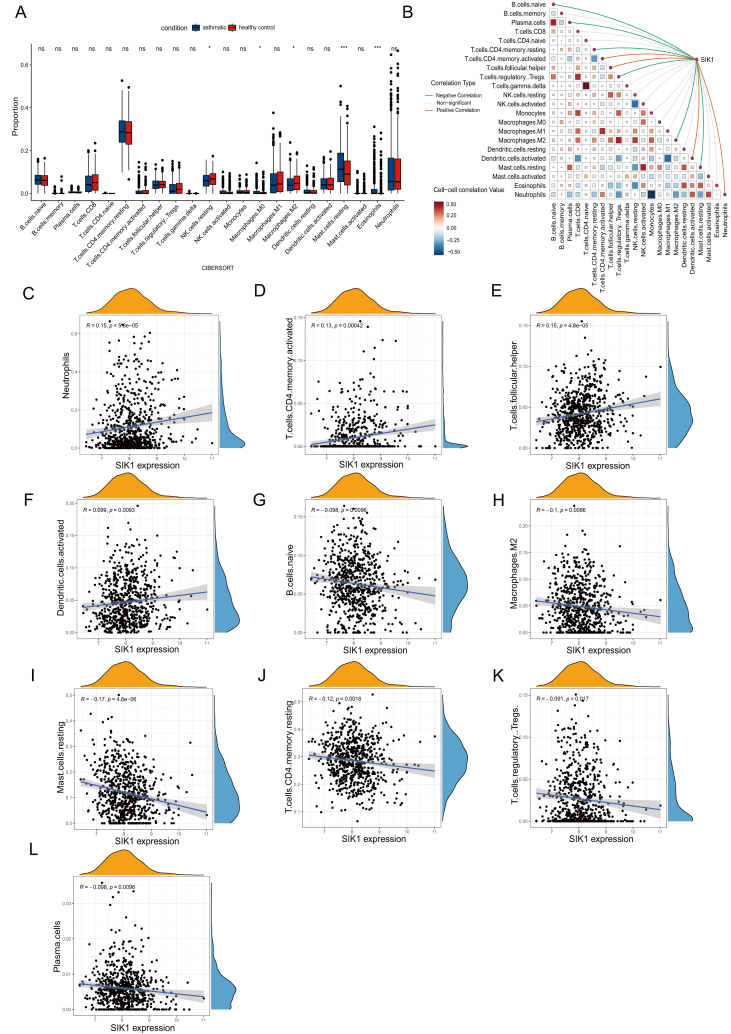
SIK1 is closely associated with multiple asthma-linked immune cells. **(A)** Bar chart of the differential fractions of infiltrated immune cells across the 22 types between control and asthma samples. **(B)** Correlation network diagram of immune cell subpopulations with gene expression. The colors of the connecting lines indicate the positive or negative correlation between SIK1 and immune cell subpopulations, while the thickness of the lines represents the strength of these correlations. The colors in the heatmap represent the strength of the correlation between different immune cells. **(C**–**L)** Correlation analysis between the core immune cells and SIK1 expression.

The results of gene enrichment analysis highlight that the immune-related pathways play a pivotal role in the pathogenesis of asthma. Consequently, the composition of immune cells in nasal airway brush samples from asthma patients was analyzed using the CIBERSORT algorithm. Compared with the healthy group, asthma patients exhibited a higher proportion of eosinophils, resting mast cells, and activated M0 macrophages, but a lower proportion of resting natural killer cells and M2 macrophages (Fig. 4A). The results of correlation analysis indicated that plasma cells were positively correlated with naive B cells, gamma delta T cells were positively correlated with naive CD4 T cells, and neutrophils were positively correlated with activated mast cells (Fig. 4B). Furthermore, the Pearson correlation coefficient showed that among the immune cell populations associated with asthma, SIK1 was positively correlated with activated CD4 memory T cells, follicular helper T cells, and activated dendritic cells. Additionally, SIK1 demonstrated a negative correlation with naive B cells, plasma cells, resting CD4 memory T cells, M2 macrophages, and resting mast cells (Fig. 4C–L).

### SIK1 is involved in the regulatory network of antiviral response and the regulation of adaptive immune response

**Figure 5:**
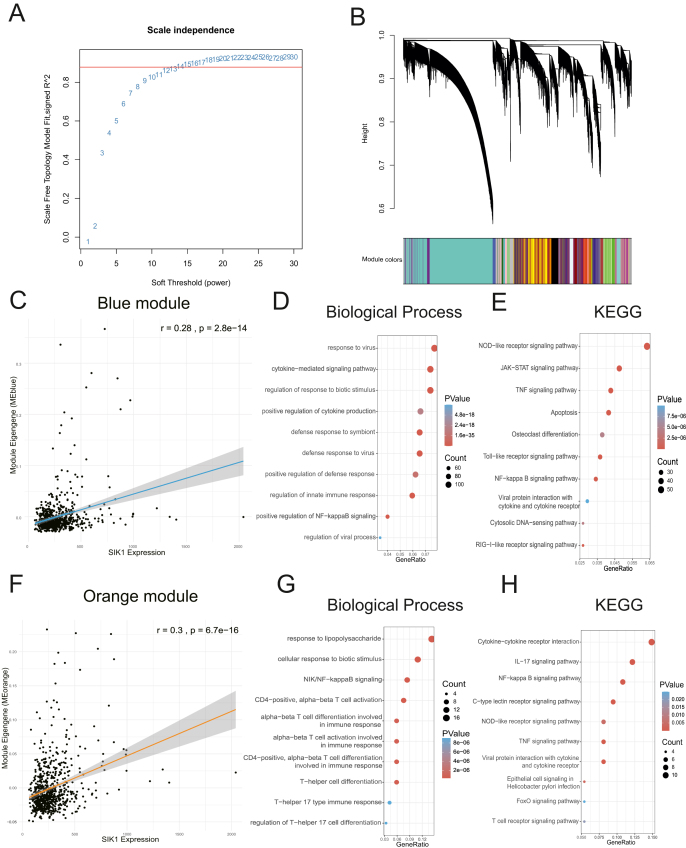
SIK1 is involved in antiviral response and adaptive immune response. **(A)** Analysis of the scale-free index for various soft-threshold powers (β). **(B)** Dendrogram of all differentially expressed genes clustered based on the measurement of dissimilarity. **(C)** The scatterplot reveals a strong positive correlation between SIK1 expression and summary (eigen gene) expression of the blue module network. *p*-values were obtained from the two-sided Pearson correlation test. **(D, E)** GO and KEGG enrichment analysis of genes in the blue module. **(F)** The scatterplot reveals a strong positive correlation between SIK1 expression and summary (eigengene) expression of the orange module network. *p*-values were obtained from the two-sided Pearson correlation test. **(G, H)** GO and KEGG enrichment analysis of genes in the orange module.

The whole transcriptome sequencing data (GSE152004) from 695 healthy and asthmatic children with nasal airway brushings were subjected to WGCNA. Topological calculations were performed using soft thresholds ranging from 1 to 20, with the optimal soft threshold determined to be 13 (Fig. 5A; Fig. S2A). The correlation matrix was then converted to an adjacency matrix and subsequently to a topological overlap matrix. The resulting topological overlap matrix enabled the application of average linkage hierarchical clustering to categorize gene modules, with a minimum of 30 genes per module. The merging of analogous gene modules led to the identification of 36 modules (Fig. 5B). SIK1 was located in the blue module (Fig. 5C), which was identified as an antiviral response module through GO enrichment analysis (Fig. 5D). KEGG enrichment analysis revealed that genes in the blue module were enriched in the nuclear factor kappa B (NF-κB) signaling pathway, JAK (Janus kinase)-signal transducer and activator of transcription (STAT) signaling pathway, Toll-like receptor signaling pathway, and other antiviral response signaling pathways (Fig. 5E). Additionally, SIK1 was strongly correlated with the orange module (*r* = 0.3; Fig. 5F), which was highly correlated with the blue module (Fig. S2B) and enriched in biological processes, such as cellular response to lipopolysaccharide, canonical NF-κB signal transduction, inflammatory response, T cell activation and differentiation, IL-17A signaling pathway, NF-κB signaling pathway, and viral protein interaction with cytokine and cytokine receptor (Fig. 5G and H). These findings suggest that SIK1 regulates signaling pathways involved in T cell activation and differentiation, potentially playing an important role in the adaptive immune response. Module-phenotype analysis showed that both the blue and orange modules were negatively correlated with asthma phenotypes (Fig. S2C), indicating that SIK1 may act as a protective factor in suppressing asthma symptoms.

### SIK1 expression is up-regulated during EV-D68-induced pulmonary inflammation in mice

**Figure 6:**
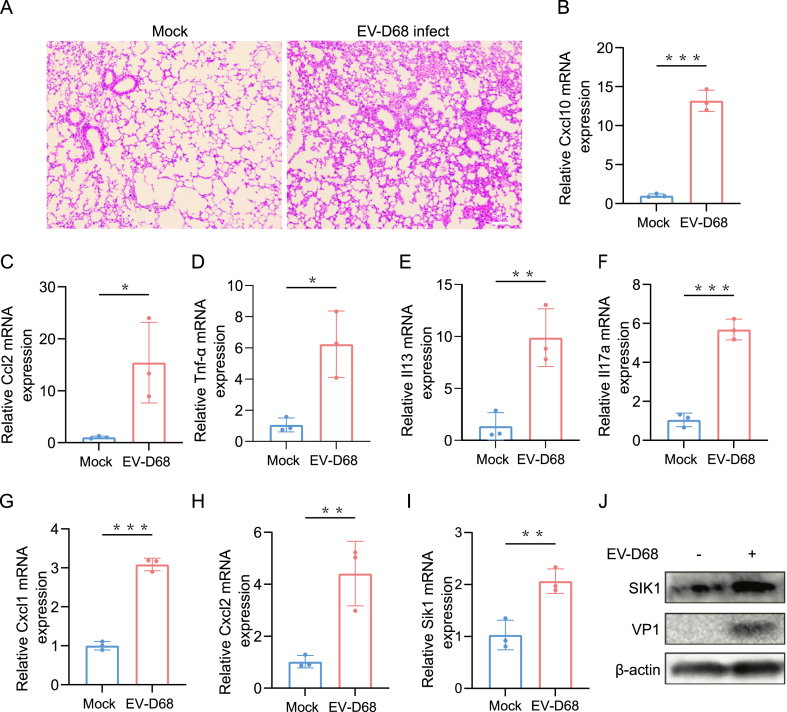
SIK1 expression is induced by EV-D68 infection *in vivo*. Eight-to-ten-week-old type I interferon receptor-deficient mice (*Ifna*^*−/−*^) were treated with EV-D68 (50 μL of 1 × 10^7^ PFU/mL viral stock for each mouse) or an equal volume of phosphate-buffered saline through the intranasal route after anesthesia. Lungs were harvested 48 h after infection and then analyzed by hematoxylin-eosin staining, quantitative PCR, and western blotting. **(A)** Images showing lung inflammation 48 h after treatment with EV-D68 or mock phosphate-buffered saline, as visualized by hematoxylin-eosin staining (scale bar: 50 mm). **(B–I)** The indicated genes were detected by quantitative PCR and normalized to GAPDH expression. Values are from three independent experiments and expressed as mean ± standard deviation. ∗*p* < 0.05, ∗∗*p* < 0.01, and ∗∗∗*p* < 0.001. **(J)** The protein expression level of SIK1 in the lungs of mice from the mock and EV-D68 groups was detected by western blotting.

We investigated the impact of EV-D68 infection on pulmonary inflammation in *Ifna*^*−/−*^ mice. The results showed that mice infected with EV-D68 displayed a notable accumulation of leukocytes and thickening of alveolar septa in lung tissues compared with the control group (Fig. 6A). The mRNA expression levels of the four inflammatory factors, C-X-C motif chemokine ligand 10 (CXCL10), chemokine ligand 2 (CCL2), TNF-α, and IL-13 were significantly increased in EV-D68-infected mice in comparison to controls (Fig. 6B–E). Of note, consistent with the above KEGG pathway enrichment analysis, the mRNA expression level of IL-17A and its downstream genes, CXCL1 and CXCL2, were also up-regulated (Fig. 6F–H). As these genes are involved in neutrophil chemotaxis, this suggests that EV-D68 infection induces neutrophilic inflammatory responses in mice.^15^ Furthermore, SIK1 mRNA and protein expression were both significantly up-regulated in EV-D68-induced lung inflammation in mice (Fig. 6I and J), suggesting that SIK1 may play a role in response to viral infection and inflammatory regulation.

### SIK1 exerts a broad-spectrum antiviral effect

**Figure 7:**
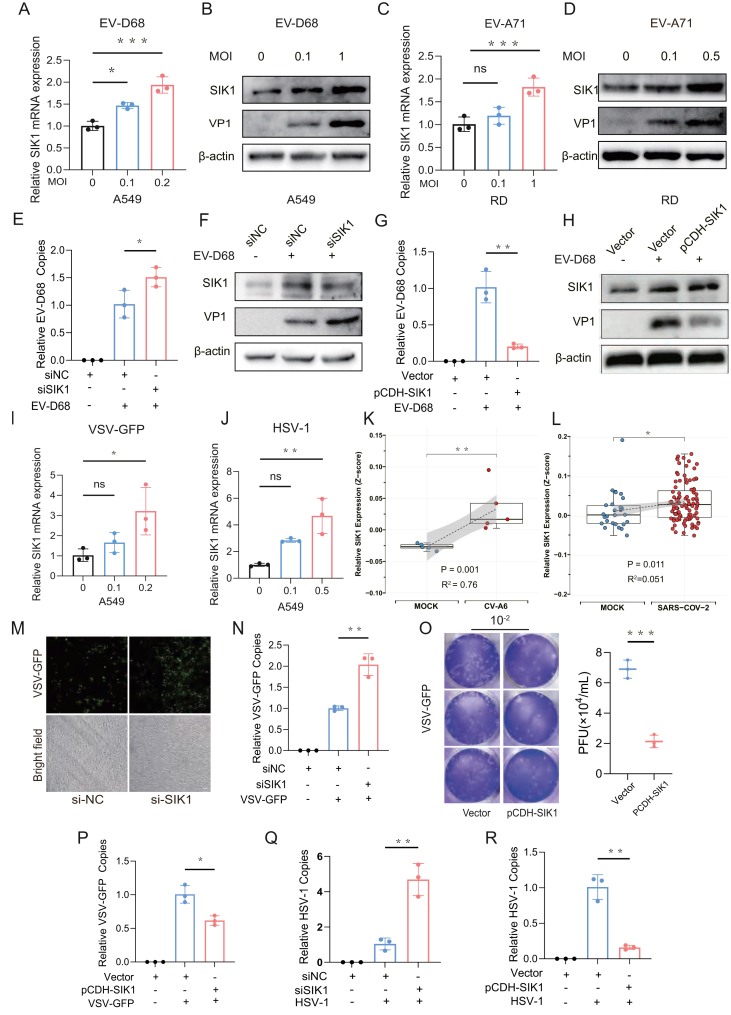
SIK1 shows antiviral effects in various viral infections. **(A, B)** A549 cells were infected with EV-D68 (MOI = 0.1 or 1) for 24 h. (A) Quantitative reverse transcription PCR (RT-qPCR) analysis of relative SIK1 mRNA expression. The results were normalized to GAPDH expression. (B) Western blotting analysis of SIK1 and EV-D68 VP1 protein expression. β-actin was used as the loading control. Values were from three independent experiments and expressed as mean ± standard deviation. **(C, D)** RD cells were infected with EV-A71 (MOI = 0.1 or 0.5) for 24 h. (C) RT-qPCR analysis of relative SIK1 mRNA expression. The results were normalized to GAPDH expression. (D) Western blotting analysis of SIK1 and EV-A71 VP1 protein expression. β-actin was used as the loading control. Values were from three independent experiments and expressed as mean ± standard deviation. **(E, F)** A549 cells were transfected with si-SIK1 or si-NC and then infected with EV-D68 (MOI = 0.5) for 24 h. (E) The relative viral RNA copy numbers were determined by RT-qPCR and normalized to GAPDH. (F) The protein expression levels of EV-D68 VP1 and SIK1 were detected by western blotting. β-actin was used as the loading control. Values were from three independent experiments and expressed as mean ± standard deviation. **(G, H)** A549 cells were transfected with plasmid pCDH-SIK1 or empty pCDH vector and then infected with EV-D68 (MOI = 0.5) for 24 h. The viral replication and protein expression level of EV-D68 VP1 and SIK1 were detected as described above. Values were from three independent experiments and expressed as mean ± standard deviation. **(I, J)** A549 cells were infected with VSV-GFP for 6 h (MOI = 0.1, 0.5) or HSV-1 for 24 h (MOI = 0.1, 0.2), and then the relative mRNA expression of SIK1 was analyzed by RT-qPCR. Values were from three independent experiments and expressed as mean ± standard deviation. **(K, L)** Box plots represent the normalized expression levels of SIK1 using Z-score normalization in GSE157103 (for CV-A6) and GSE243200 (for SARS-COV-2) datasets. SIK1 expression correlation was analyzed using Spearman's method. **(M, N)** A549 cells were transfected with si-SIK1 or si-NC and then infected with VSV-GFP (MOI = 0.5) for 12 h. The replication of VSV-GFP was visualized by immunofluorescence microscopy (scale bar: 50 μm), and VSV-GFP RNA synthesis was determined by RT-qPCR analysis. Values were from three independent experiments and expressed as mean ± standard deviation. **(O, P)** A549 cells were transfected with plasmid pCDH-SIK1 or empty pCDH vector and then infected with VSV-GFP (MOI = 0.5) for 12 h. To detect viral replication by quantitative PCR, viral titers were determined by plaque assay as described in the methods section, and viral RNA synthesis was determined by RT-qPCR analysis. Values were from three independent experiments and expressed as mean ± standard deviation. **(Q, R)** SIK1 was interfered with by si-SIK1 or overexpressed via transfection of plasmid pCDH-SIK1 in A549 cells, and then the cells were infected with HSV-1. Viral replication was determined by RT-qPCR. Values were from three independent experiments and expressed as mean ± standard deviation. ∗*p* < 0.05, ∗∗*p* < 0.01, and ∗∗∗*p* < 0.001; ns, non-significant.

To test the effect of SIK1 in EV-D68 infection, the expression pattern of SIK1 was first assessed in A549 cells infected with EV-D68. The results demonstrated a dose-dependent increase in SIK1 expression at both protein and mRNA levels (Fig. 7A and B). Similar results were obtained in RD cells infected with EV-A71, an orthopoxvirus of EV-D68 (Fig. 7C and D). To substantiate the involvement of SIK1 in the antiviral response, we silenced SIK1 in the A549 cell line using siRNA and then infected the cells with EV-D68. The results showed a significant increase in EV-D68 mRNA and VP1 protein levels (Fig. 7E and F). Conversely, EV-D68 VP1 expression was greatly inhibited in A549 cells upon overexpression of SIK1 (Fig. 7G and H).

We next explored whether SIK1 played a broad-spectrum role in antiviral immunity. A549 cells were infected with VSV-GFP or HSV-1, both of which substantially increased SIK1 transcription (Fig. 7I and J). Additionally, an analysis of the GSE157103 and GSE243200 datasets revealed that SIK1 expression was also significantly elevated following SARS-CoV-2 or Coxsackie virus A6 (CV-A6) infection (Fig. 7K and L). These findings suggest that multiple viruses can significantly activate SIK1 expression. Further, more GFP fluorescence spots and increased VSV-GFP copies were observed in SIK1 knockdown A549 cells compared with the control group (Fig. 7M and N). Conversely, overexpression of SIK1 in A549 cells resulted in a marked reduction in VSV-GFP plaque-forming units (PFU) and VSV-GFP copies (Fig. 7O and P). Similar results were observed in SIK1 knocked-down or SIK1 overexpressed A549 cells during HSV-1 infection (Fig. 7Q and R). These data collectively indicate that SIK1 has a broad-spectrum antiviral effect.

### Activation of SIK1 alleviates EV-D68-induced asthma exacerbation in mice

**Figure 8:**
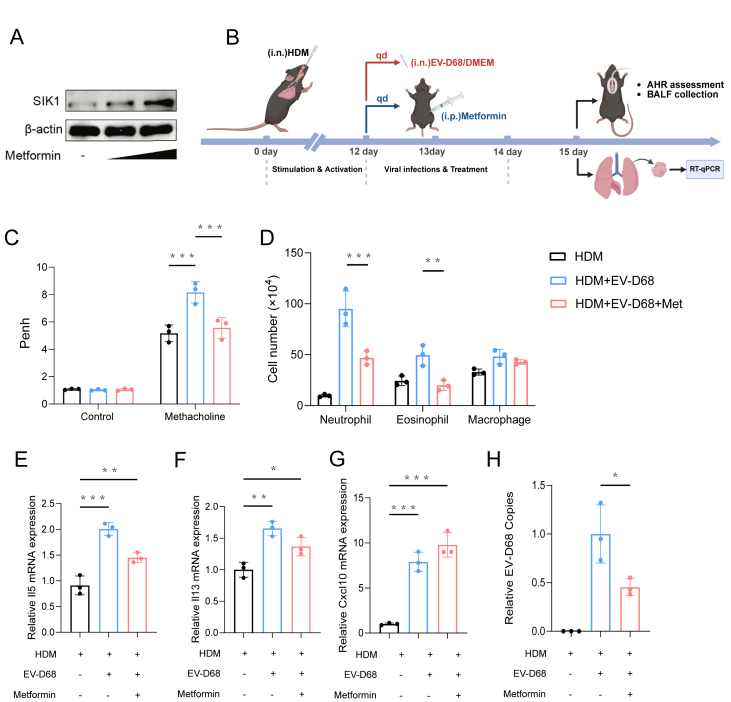
Metformin-mediated activation of SIK1 protects against EV-D68-driven asthma exacerbation in house dust mite (HDM)-sensitized mice. **(A)** C57BL/6 mice (6–8 weeks) were administered metformin at doses of 100 mg/kg or 250 mg/kg once daily via intraperitoneal injection on day 1 and day 2. On day 3, lung tissues were collected, and the protein level of SIK1 was determined by western blotting analysis. **(B)** Experimental timeline. C57BL/6 mice (6–8 weeks) were intranasally sensitized with 250 μg kg^−1^ HDM extract on day 0 and challenged daily with the same dose on days 7–11. On days 12–13, animals received EV-D68 (1 × 10^6^ PFU/kg) or DMEM (vehicle) intranasally. Metformin (100 mg/kg, intraperitoneal) was administered once daily on days 12–14. Airway hyper-responsiveness measurements and broncho-alveolar lavage fluid (BALF) collection were performed on day 15; lung tissue was used for quantitative PCR analyses. **(C)** Airway responsiveness to increasing doses of methacholine. **(D)** Differential cell counts of BALF by Wright-Giemsa staining. **(E**–**H)** The indicated genes were detected by quantitative PCR and normalized to GAPDH expression. Values were from three independent experiments and expressed as mean ± standard deviation. ∗*p* < 0.05, ∗∗*p* < 0.01, and ∗∗∗*p* < 0.001.

To further evaluate the protective effect of SIK1 in EV-D68-induced asthma exacerbation, we first assessed whether metformin, a known SIK1 activator,^16^^,^^17^ could enhance SIK1 expression in the lungs. Lung tissues were collected 72 h after metformin administration and subjected to western blotting analysis. The results showed a dose-dependent increase in SIK1 protein levels in the metformin-treated group compared with the control (Fig. 8A).

Subsequently, we established a mouse model of EV-D68-associated asthma exacerbation using HDM sensitization followed by EV-D68 infection (Fig. 8B). As expected, co-treatment with HDM and EV-D68 significantly increased the number of neutrophils and eosinophils in BALF, along with enhanced airway hyper-responsiveness (Fig. 8C and D). Metformin treatment markedly alleviated these responses, as evidenced by reduced BALF neutrophil and eosinophil counts and improved airway hyper-responsiveness.

At the molecular level, metformin administration resulted in significant down-regulation of type 2 cytokines IL-5 and IL-13, and a concurrent up-regulation of the antiviral chemokine Cxcl10 (Fig. 8E–G). These immunological changes were accompanied by a notable reduction in EV-D68 genomic copy numbers in the lung (Fig. 8H). Collectively, these data indicate that SIK1 activation via metformin mitigates EV-D68-induced airway inflammation and hyper-responsiveness, likely through a dual mechanism involving suppression of type 2 inflammation and enhancement of antiviral responses.

## Discussion

Prior research suggests that EV-D68 infection could induce neutrophil-mediated airway inflammation and allergic responses through the activation of IL-17A.^15^ In this study, IL-17A showed significant enrichment and up-regulation in KEGG enrichment analysis of coDEGs and subsequent GSEA enrichment analysis of GSE184488 and GSE143303. Another study reviewed that IL-17A increases the expression of chemokines, such as CXCL1 and CXCL2, which bind to CXCR receptors. Upon release, these chemokines bind to CXCR receptors on neutrophils, directing their migration along a gradient to sites of inflammation, which activates neutrophilic asthma.^18^ We verified using EV-D68 infection animal models that IL-17A and its downstream chemokines CXCL1 and CXCL2 were significantly activated by EV-D68 infection. Taken together, it is reasonable to hypothesize that EV-D68 infection activates an asthma-related immune response, further aggravating asthma. However, the relative molecular mechanism is still deficient.

Subsequently, we comprehensively assessed coDEGs using multiple machine learning algorithms and identified SIK1 as an EVD-68 infection-associated signature gene in the airways of patients with asthma. Further analysis confirmed SIK1 as a key gene in the antiviral regulatory network, and *in vitro* experiments proved its inhibitory role in the intrinsic immune response against a wide range of viruses. SIK1 is a serine–threonine kinase belonging to the AMP-activated protein kinase (AMPK) family and is involved in a number of cellular and physiological processes.^19^ It continues to phosphorylate downstream targets, including transcriptional cofactors CREB-regulated transcription coactivator 1/2/3 (CRTC1//2/3) and class IIa histone deacetylase (HDAC).^19^, ^20^, ^21^

Viral infections are often accompanied by metabolic reprogramming, whereby viruses can hijack the host's lipid and glucose metabolic processes to provide energy and raw materials for their own replication and assembly processes.^22^ Previous studies have demonstrated the important regulatory role of SIK1 in metabolic homeostasis, inhibiting gluconeogenic pathways and lipogenesis.^21^^,^^23^ Therefore, when activated in viral infections, SIK1 may enhance innate antiviral immunity by optimizing host metabolic reprogramming and limiting viral replication. In addition, a modest inflammatory response is an important component of intrinsic immunity, which helps to attract immune cells to the site of infection and clear the pathogen. Therefore, by activating pro-inflammatory signaling,^24^^,^^25^ SIK1 helps host cells to rapidly initiate effective antiviral defense mechanisms.

SIK1 is also known to facilitate communication between T cells by phosphorylating the ubiquitin 1 (Panx1) channel at the S205 site, which is essential for reducing the severity of airway inflammation.^26^ Combined with the results of immune infiltration analysis and WGCNA module enrichment analysis, we further determined that SIK1 played an important role in adaptive immunity through activation of T cells. Previous experiments have demonstrated that SIK1 endogenous activity is required to maintain regulatory T cell survival.^27^ In the pathological development of asthma, T cells are not only directly involved in the immune response but also exert an influence on the inflammatory response by interacting with immune cells, such as mast cells.^28^ Recently, it has been proven that pre-existing asthmatic inflammation in the lower airways may modify the immune response to viral infection, leading to delayed viral clearance, persistent virus-induced inflammation, and amplification of asthma exacerbations.^29^ Our results suggest that positive regulation of T cells by SIK1 contributes to the simultaneous control of airway inflammation and viral load, which plays an important protective role against EV-D68-induced asthma.

A few genes have been reported to have a protective role in asthma caused by viral infections. For example, carcinoembryonic antigen-related cell adhesion molecule 3 (CEACAM3) decreases asthma exacerbations and modulates latent respiratory syncytial virus infection in children,^30^ while IL-17A protects against rhinovirus-induced asthma by inhibiting rhinovirus replication in epithelial cells.^31^ However, reports of genes that inhibit asthma development through antiviral functions are still rare. In this study, we demonstrate for the first time that SIK1 possesses potential broad antiviral activity and shields the airway from EV-D68-induced asthma. Because an inadequate antiviral response is a key driver of virus-mediated exacerbation of asthma,^32^ reinforcing these pathways is increasingly viewed as a promising therapeutic avenue.^33^

Our *in vivo* findings underscore the importance of achieving balanced immune modulation—attenuating pathogenic inflammation while preserving essential antiviral defenses. In EV-D68-infected mice, pharmacological activation of SIK1 using metformin was associated with a reduction in type 2 cytokines IL-5 and IL-13, alongside an increase in the antiviral chemokine CXCL10. These immunological changes corresponded with decreased airway inflammation and reduced airway hyper-responsiveness compared with untreated controls. This suggests that SIK1 activation may simultaneously down-regulate type 2-driven eosinophilic inflammation and enhance innate antiviral responses, contributing to improved disease outcomes. Given that impaired antiviral immunity is a key contributor to virus-induced asthma exacerbations, targeting host antiviral pathways may offer a complementary strategy to traditional anti-inflammatory therapies. Our data support SIK1 as a potential therapeutic target in this context. Further studies employing selective SIK1 modulators or genetic models will be necessary to elucidate its precise immunomodulatory mechanisms during viral infection and asthma exacerbations.

## CRediT authorship contribution statement

**Juntong Liu:** Writing – original draft, Visualization, Investigation, Conceptualization. **Yue Wang:** Writing – review & editing, Investigation, Conceptualization. **Lingyun Zou:** Writing – original draft, Visualization. **Xinyue Han:** Investigation, Methodology, Validation. **Mingqi Lv:** Project administration, Funding acquisition. **Xichuan Deng:** Project administration. **Jingjing Liao:** Data curation. **Guangchao Zang:** Software. **Lei Xu:** Writing – review & editing. **Tianle Gu:** Writing – review & editing. **Nan Lu:** Supervision, Project administration. **Guangyuan Zhang:** Project administration, Funding acquisition.

## Ethics declaration

All the animal experiments were conducted according to the protocol approved by the Ethics Committee of Chongqing Medical University (approval number: IACUC-CQMU-2024-0669).

## Funding

This work was supported by grants from the 10.13039/501100001809National Natural Science Foundation of China (No. 31600139), the 10.13039/501100007957Chongqing Municipal Education Commission of China (No. CSTB2024NSCQ-KJFZMSX0067), the Project of Undergraduates Innovating Experiment and the Project of Tutorial System of Excellent Medical Undergraduate in Lab Teaching and Management Center of Chongqing Medical University (China) (No. 202211, S202410631068, 202510631005, LTMCMTS202310, LTMCMTS202311, LTMCMTS202312, LTMCMTS202458, LTMCMTS202459, LTMCMTS202460, LTMCMTS202461), the Program for Youth Innovation in Future Medicine, Chongqing Medical University (China) (No. W0160), the Chongqing Medical Scientific Research Project (Joint Project of Chongqing Health Commission and Science and Technology Bureau (No. 2024ZDXM011), and the 10.13039/501100005230Natural Science Foundation of Chongqing, China (No. CSTB2023NSCQ-MSX0237).

## Conflict of interests

The authors declared no competing interests.

## Appendix A Supplementary data

Figure S1 Gene set enrichment analysis of EV-D68 infection and asthma datasets. Figure S1

Figure S2 Weighted gene co-expression network analysis of the asthma dataset. **(A)** Analysis of the mean connectivity for various soft-threshold powers (β). **(B)** Tree diagram clustering of modules. **(C)** Heatmap of correlation between modules and traits. Figure S2

Table S1 Summary of the PCR primers and the siRNA sequences used in the study. Table S1

Table S2 Details on the relevant single-nucleotide polymorphism parameters. Table S2
