# Supplementary figures and images for: Salt-inducible kinase 1 is a key gene in suppressing EVD68-induced asthma by modulating antiviral immunity
Source: Genes Dis. 2025 Sep 10;13(3):101845. doi: 10.1016/j.gendis.2025.101845 (PMC12886539; doi:10.1016/j.gendis.2025.101845)

A

# GSEA GSE184488

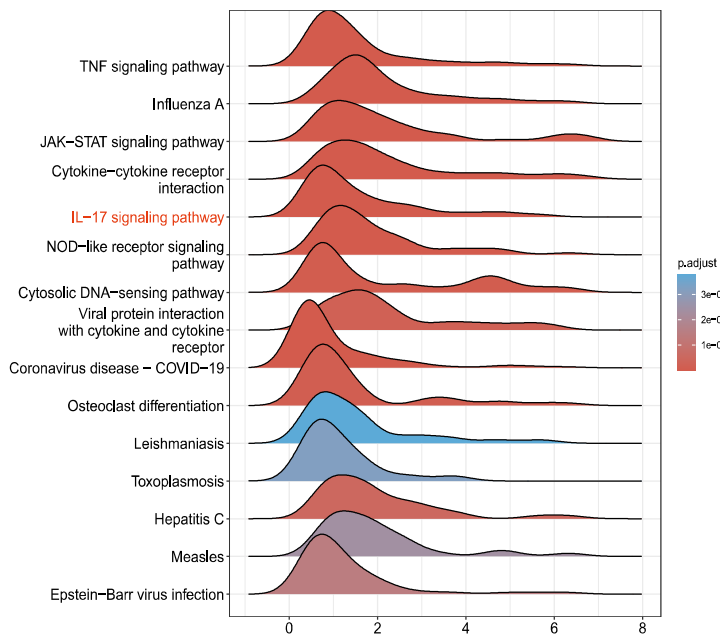

B

# GSEA GSE143303

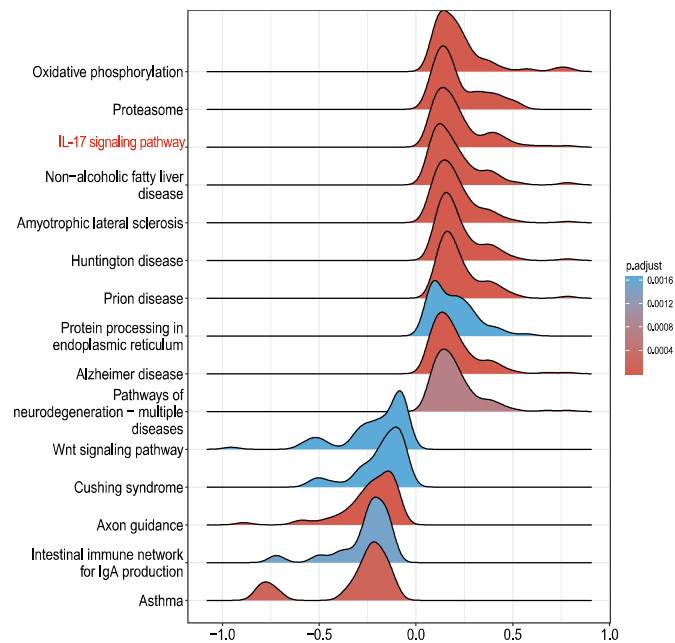

Supplement: Figure S1 — Gene set enrichment analysis of EV-D68 infection and asthma datasets. [file mmc1.pdf]

A

Mean connectivity

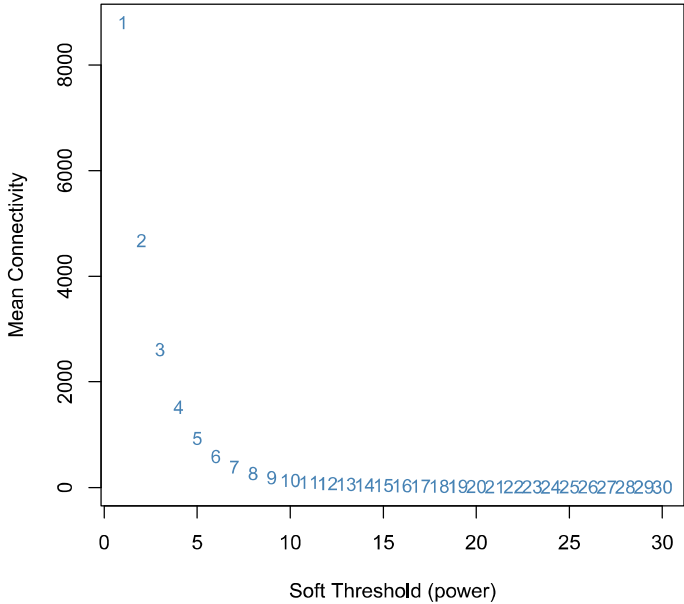

B

Module clustering

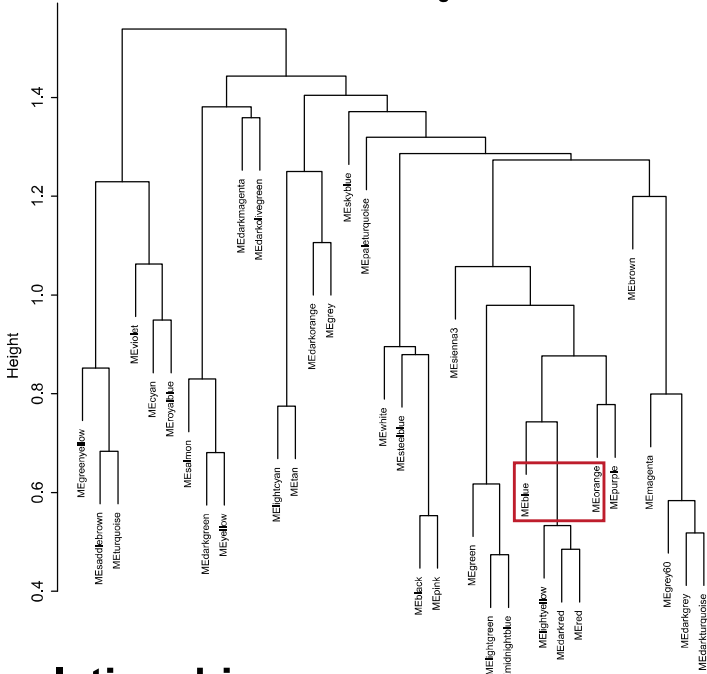

C

Module-trait relationships

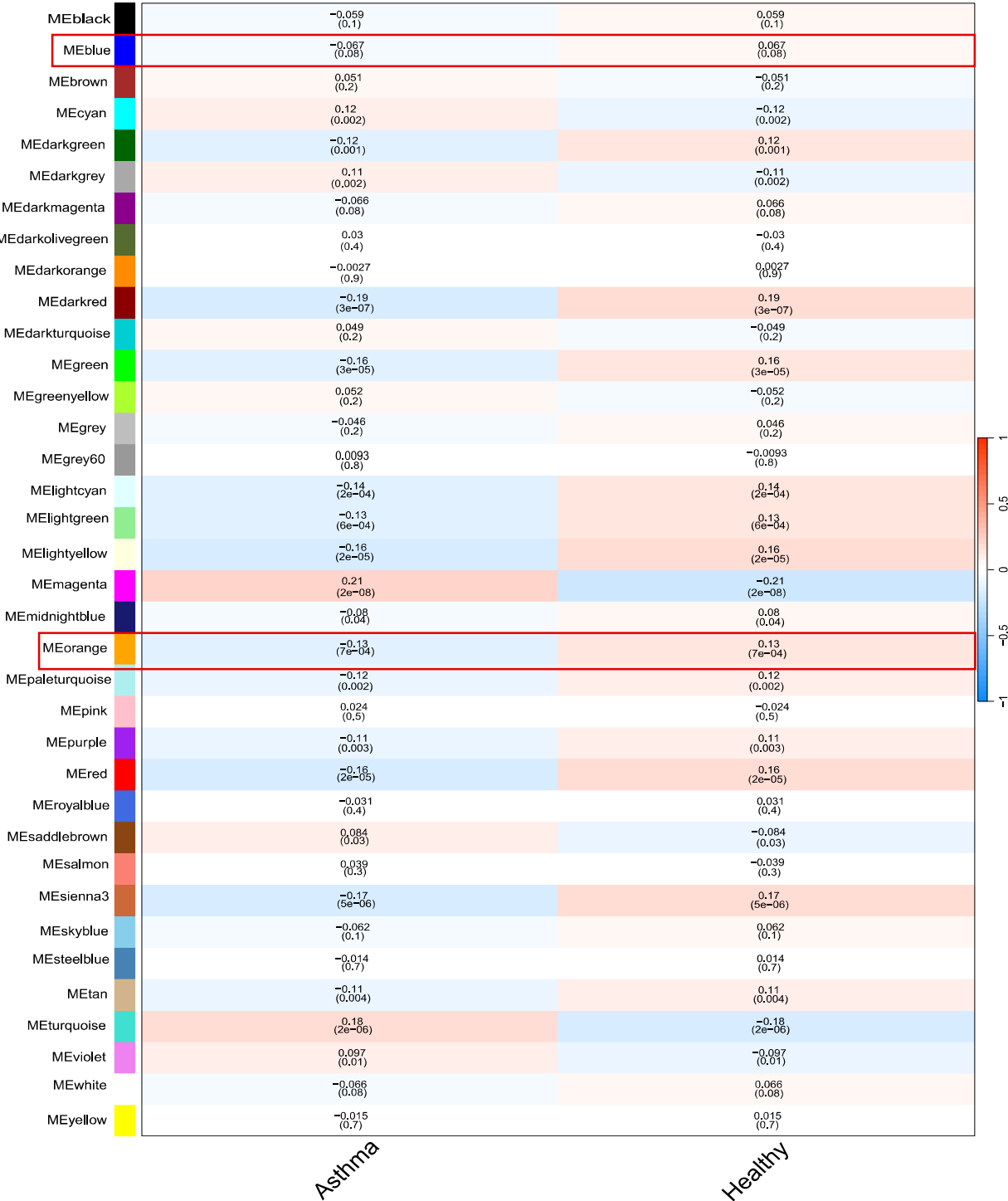

Supplement: Figure S2 — Weighted gene co-expression network analysis of the asthma dataset. (A) Analysis of the mean connectivity for various soft-threshold powers (β). (B) Tree diagram clustering of modules. (C) Heatmap of correlation between modules and traits. [file mmc2.pdf]
